# Non‐Volatile Phase Modulation with Ultralow Energy Consumption Enabled by 2D Ferroelectric/TMD Heterostructures

**DOI:** 10.1002/advs.202520795

**Published:** 2026-01-28

**Authors:** Lalit Singh, Shi Guo, Yuhui Yang, Sholehin Juperi, Rui Yu, Xiangxin Gong, Jeremy Leong, Sung‐Gyu Lee, Qingyun Wu, Lay Kee Ang, Sang Hoon Chae

**Affiliations:** ^1^ School of Electrical and Electronic Engineering Nanyang Technological University Singapore Singapore; ^2^ CNRS‐International‐NTU‐Thales Research Alliance (CINTRA) Singapore Singapore; ^3^ Science, Mathematics and Technology Singapore University of Technology and Design (SUTD) Singapore Singapore; ^4^ School of Materials Science and Engineering Nanyang Technological University Singapore Singapore

**Keywords:** Compactness, multi‐level memory, ferroelectricity, low‐loss, non‐volatile, Phase modulation, ultralow energy consumption

## Abstract

Achieving non‐volatile, low‐loss phase modulation with ultra‐low energy consumption remains a challenge in photonic in‐memory computing. Inspired by electrical memory technologies, mechanisms such as ion‐migration, phase change transitions, and ferroelectric polarization have been explored in photonic platforms for memory functions. However, existing materials typically require large device footprints to achieve effective optical index tuning, leading to increased insertion loss and energy consumption. Here, we demonstrate a compact non‐volatile phase modulator by incorporating 2D ferroelectric CuInP_2_S_6_ (CIPS) into a WS_2_/CIPS/graphene heterostructure, integrated on a SiN microring resonator. This vertical configuration leverages Cu^+^‐induced polarization in CIPS to electrostatically tune the refractive index of WS_2_ without introducing additional optical loss or static power consumption. The intralayer Cu^+^‐mediated ferroelectric switching (free from domain wall motion) and high dielectric constant enable the device to operate with an ultra‐low switching energy of 2.5 pJ per cycle, a fast write speed of 5 V/µs, and an insertion loss of 0.2 dB. The device further shows stable multi‐level (8‐bit) memory, with projected retention beyond 10 years. We showcase its potential in photonic in‐memory computing by implementing the modulator within an optical neural network, achieving 92% accuracy on the MNIST handwritten digit recognition, establishing new avenues for hardware‐accelerated neural networks.

## Graphical abstract

We demonstrate a hybrid WS_2_/CuInP_2_S_6_/graphene heterostructure integrated on a silicon nitride microring resonator for non‐volatile optical phase modulation with ultra‐low energy consumption and low insertion loss. While CIPS alone does not provide efficient optical index modulation, the engineered proposed device structure converts ferroelctric domain switching into robust, low‐loss, and non‐volatile phase tunning.

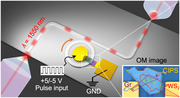

## 1 Introduction

The growing demand for real‐time artificial intelligence (AI) applications ranging from autonomous vehicles, edge computing to cloud‐based language models has placed unprecedented pressure on existing electronic hardware [1, 2, 3, 4, 5, 6]. While custom electronic accelerators such as graphical processing unit and application‐specific integrated circuit have improved artificial neural network performance, they remain constrained by fundamental limitations of electronic charge transport, including Joule heating, interconnect bottlenecks, and poor scalability in data movement [7, 8, 9]. Optical neural networks (ONNs), built on photonic integrated circuits (PICs), can perform matrix‐vector multiplications at the speed of light, promising orders‐of‐magnitude improvements in throughput (high bandwidth), energy efficiency and isolation to electromagnetic interference over their electronic counterparts [5, 6, 7, 10, 11, 12, 13, 14]. Integrated photonics allows parallel data processing, ultra‐fast modulation, and low‐latency signal propagation using light.

Among integrated photonic platforms, silicon nitride (SiN) stands out for its ultra‐low loss, high quality factor (Q), absence of two‐photon absorption at telecom wavelengths, and wide thermal compatibility [15, 16], but its passive nature necessitates functional material integration for active modulation. In this context, traditional tuning mechanisms, such as thermo‐optic and carrier‐injection effects, are inherently volatile and requires continuous power to maintain their states and contributing to static energy consumption [17, 18, 19]. This volatility fundamentally limits the scalability and energy efficiency of integrated photonics. Despite advances in photonic integration, the lack of low‐loss, non‐volatile optical phase shifters with low energy consumption fundamentally limits the deployment of scalable photonic in‐memory computing architectures.

Non‐volatile memory functions have been demonstrated using bulk materials such as BaTiO_3_, VO_2_, HfZrO etc. via phase change, ferroelectricity, charge trapping, or ion migration [9, 11, 20, 21, 22, 23, 24]. However, these materials often face issues such CMOS compatible fabrication, large footprints and optical insertion losses [25]. 2D van der Waals (vdW) materials such as monolayer tungsten diselenide (WS_2_) offer seamless integration with integrated photonics and have shown considerable promise as electro‐optic materials for efficient phase modulation with minimal optical absorption loss [26, 27], but they lacks the non‐volatile functionality. 2D materials such as CuInP_2_S_6_ (CIPS), In_2_Se_3_, SnS, and CuCrP_2_S_6_ has shown room‐temperature ferroelectricity, offering promising paths toward non‐volatile functionality [28, 29, 30, 31, 32, 33, 34]. The layered dielectric ferroelectric, CIPS, has shown robust out‐of‐plane polarization at room‐temperature, offering a promising route toward integrated non‐volatile photonic memory elements [35, 36, 37, 38]. While ferroelectricity offers non‐volatility, its translation into effective optical phase modulation requires careful engineering of the waveguide‐material interface. By combining transparent 2D TMDs, capable of large index modulation with out‐of‐plane ferroelectric materials like CIPS, we can achieve electrical control of the refractive index without introducing additional optical loss [39]. This synergistic integration enables compact, low‐loss, and non‐volatile phase modulation, offering a scalable path for photonic in‐memory computing.

In this work, we demonstrate a non‐volatile phase modulation using a WS_2_/CIPS/graphene (Gr) heterostructure integrated onto a SiN photonic platform, aimed at translating ferroelectric switching into effective, low‐loss optical modulation. The ferroelectric switching in CIPS originates from the intralayer Cu^+^ displacement, which electrostatically dopes the adjacent WS_2_, resulting in index change and hence non‐volatile optical phase shift behavior. The device operates at an ultra‐low switching energy of ∼2.5 pJ per cycle, with fast write speeds (5 V/µs), insertion loss below 0.2 dB, and stable multi‐level (8‐bit) retention projected for more than 10 years. Finally, we validate its functionality in memory computing by implementing an ONN for handwritten digit recognition using the modified National Institute of Standards and Technology (MNIST) dataset, achieving over 92% accuracy. This work establishes a promising platform for an energy‐efficient non‐volatile phase modulator for in‐memory photonic computing.

## 2 Results

### 2.1 2D Ferroelectric CuInP_2_S_6_

**FIGURE 1:**
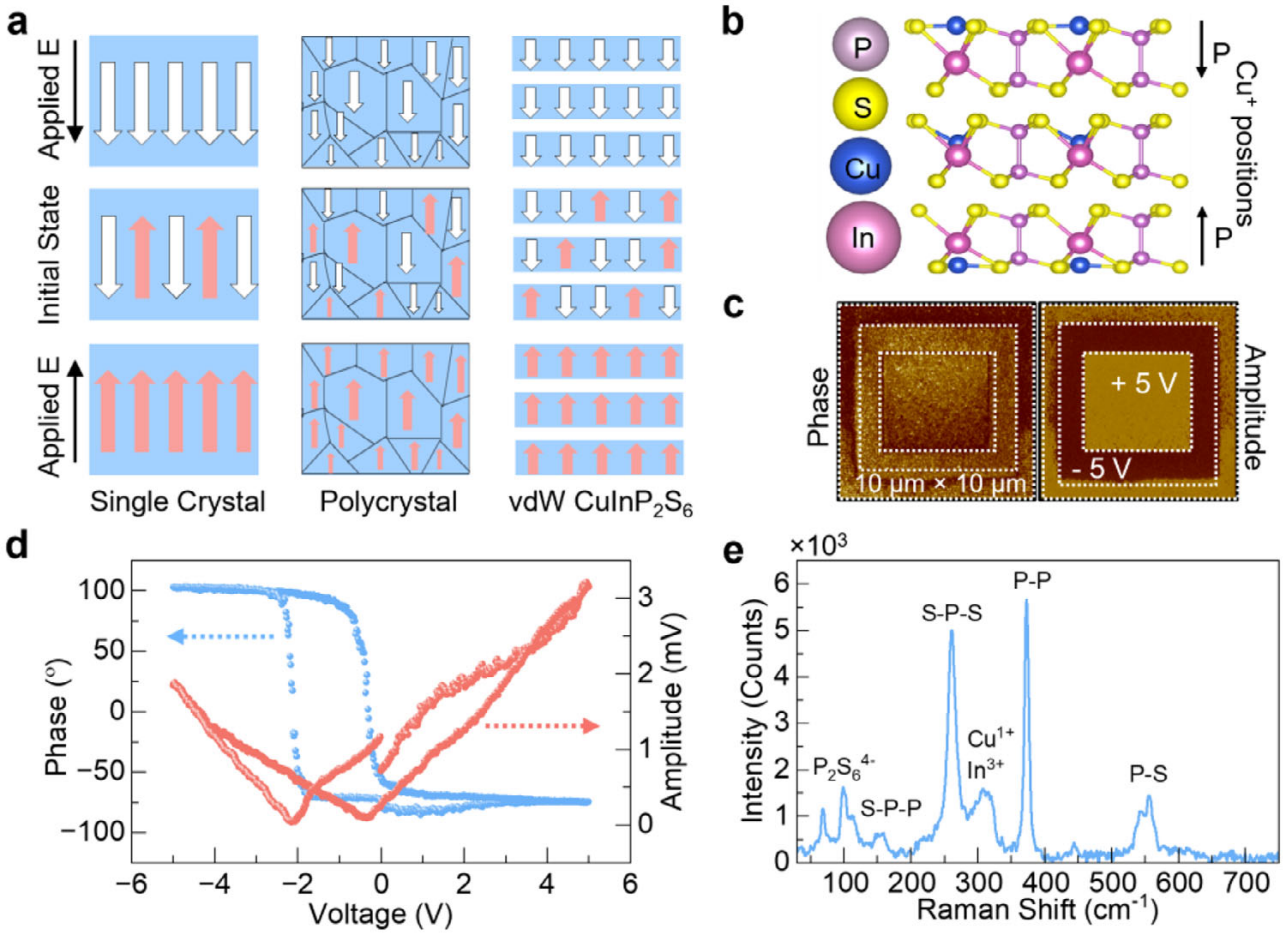
Confirmation of ferroelectric domain switching and structural characterization of CIPS. (a) Schematic illustrating various mechanisms of ferroelectric domain switching. (b) atomic structure depicting the position of Cu ions within a monolayer of CIPS. (c) Ferroelectricity domain patterning by piezoresponse force microscopy (PFM), showing amplitude and phase images that indicate domain reorientation under an applied electric field. (d) Local amplitude and phase response measured at a single‐point in CIPS. (e) Raman spectrum of the CIPS material confirming its vibrational modes.

To understand the origin of non‐volatile phase modulation using our proposed WS_2_/CIPS/Gr heterostructure, we begin by examining the ferroelectric properties of CIPS. Unlike conventional ferroelectric materials, CIPS is a layered vdW compound exhibiting robust out‐of‐plane polarization at room temperature, enabled by the displacement of Cu ions within its lattice. Figure 1a illustrates key ferroelectric switching mechanisms in different types of materials, including single‐crystal, polycrystal, and domains in vdW materials, highlighting how CIPS differs by offering switchable dipoles without requiring bulk displacement or interfacial strain. The atomic structure of monolayer CIPS (Figure 1b) shows the position of mobile Cu^+^ ions, which are responsible for domain inversion under a vertical electric field. In CIPS, the Cu^+^ ion occupies one of two inequivalent off‐center positions along the layer normal within a sulfur cage. An out‐of‐plane electric field biases Cu^+^ to hop to the opposite site, altering the nearest‐neighbor Cu‐S distances and reversing the polarization.

To experimentally confirm ferroelectricity, we performed piezoresponse force microscopy (PFM), revealing clear domain contrast in both amplitude and phase (Figure 1c). We patterned nested square domains by tip‐bias poling: 5 V over a 10 µm × 10 µm area (multiple passes) to uniformly pole the region, followed by ‐5 V over a 7.5 µm × 7.5 µm inner square, and a final 5 V over a 5 µm × 5 µm core. Subsequent PFM imaging exhibits strong amplitude contrast and ∼180° phase reversal between the squares, evidencing controllable domain inversion. We further assessed localized switching behavior using single‐point switching (Figure 1d). On sweeping the tip bias from ‐4 to +4 V and back, the PFM phase exhibits a hysteretic reversal, while the amplitude shows the characteristic butterfly loop‐clear signatures of reversible polarization switching in the 100 nm CIPS film. Raman spectroscopy (Figure 1e) further validates the material composition and vibrational modes characteristic of CIPS. Spectra were acquired at room temperature with 532 nm excitation, low power to avoid heating, and Lorentzian fits to extract peak positions. These foundational measurements confirm CIPS compositions and establish the non‐volatile switching mechanism used in our device and pave the way for understanding its integration in photonic phase modulation.

**FIGURE 2:**
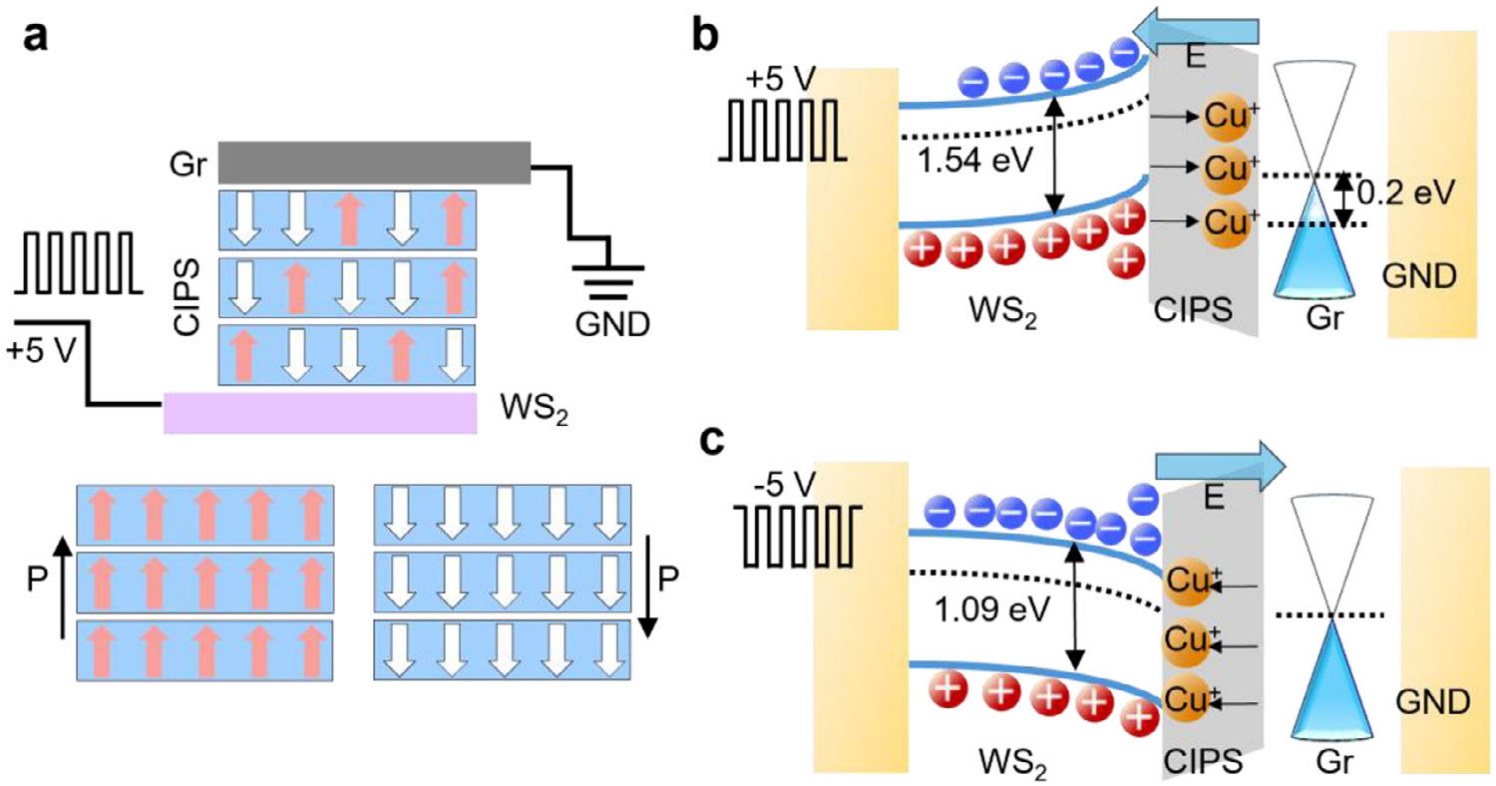
Heterostructure design and DFT validation of polarization‐coupled bandgap shifts in WS_2_ and Gr. (a) Schematic of the proposed heterostructure, where the CIPS layer is sandwiched between two electrodes: monolayer Gr and WS_2_. The illustration depicts how the application of voltage pulses modulates the domain orientation or Cu ion positions within CIPS, thereby affecting the bandgap of the adjacent WS_2_ layer. Confirmed by DFT calculations (Figure S2), the variation in the WS_2_ bandgap is a function of Cu ion positions within CIPS, and the doping changes in 1L Gr (b) when Cu^+^ is aligned near the Gr on application of positive pulses, (c) when Cu^+^ is aligned near the WS_2_ on application of negative pulses.

Building on the confirmation of out‐of‐plane ferroelectricity in CIPS, we designed a heterostructure where CIPS is vertically sandwiched between Gr and monolayer WS_2_ electrodes. This configuration was chosen to minimize optical loss and maximize phase modulation efficiency. WS_2_ exhibits a strong refractive index tunability under electrostatic gating, and provides a transparent, low‐loss contact. As shown in Figure 2a, applying voltage pulses reorients Cu^+^ ions within the CIPS layer, modulating the local electric field and thereby altering the band alignment and bandgap of the adjacent WS_2_. This effect is supported by DFT calculations (Figure S2), which show a bandgap shift in WS_2_ depending on the Cu^+^ position. Specifically, when Cu^+^ ions migrate toward Gr under positive pulses (Figure 2b), or toward WS_2_ under negative pulses (Figure 2c), the band structure of the adjacent layers responds accordingly, enabling tunable optical modulation in the waveguide. Using DFT calculations, we found that the position of Cu^+^ ions within the vdW layer of CIPS significantly alters the band structure of both adjacent WS_2_ and Gr. The calculated band structure is shown in Figure S2. The refractive index changes significantly, up to Δ*n *≈ 0.3, as derived from the calculated band structure using the following relation:

(1) $${\left(\frac{n_{2}}{n_{1}}\right)}^{4}=\frac{E_{g1}}{E_{g2}}\;$$

When Cu^+^ ions are located away from the WS_2_ interface, the calculated bandgap is ∼1.54 eV. In contrast, when Cu^+^ ions migrate closer to the WS_2_ side, the bandgap is reduced to ∼1.09 eV, indicating a strong electrostatic influence. For Gr, the effect is weaker but still notable: the Dirac point remains unchanged when Cu^+^ ions are nearby and shifts to ∼0.2 eV when they are farther away. This ion displacement under applied bias gives rise to ferroelectric behavior in CIPS and results in electrostatic doping of both electrodes. To further confirm the index change due to the CIPS polarization effect, the surface bound polarization induced carriers are calculated (Section S4), and subsequently this increased charge carriers changesthe permittivity and index of the WS_2_.

### 2.2 Nonvolatile Phase Modulator Using WS_2_/CIPS/Gr

**FIGURE 3:**
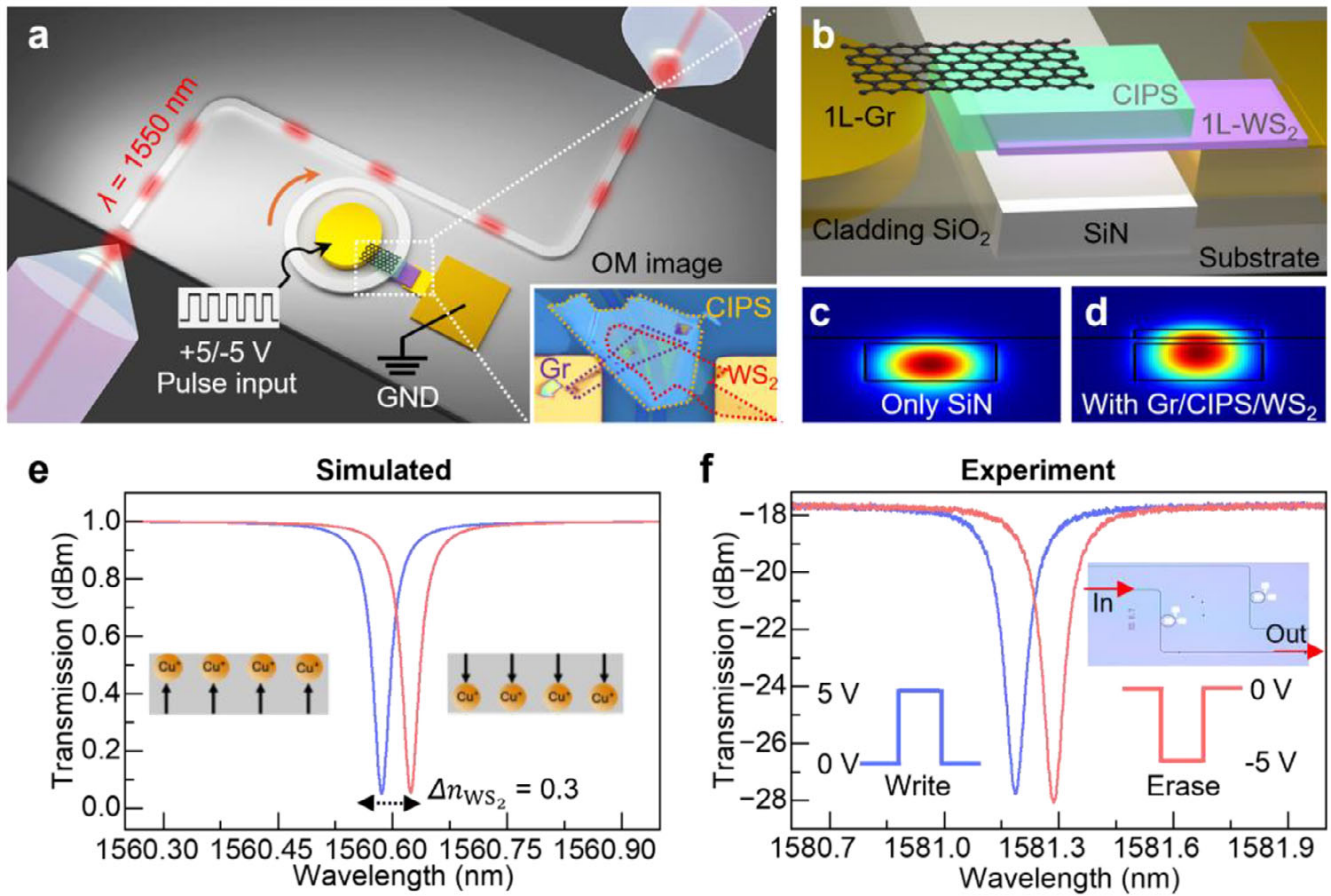
Device structure and validation of phase modulation enabled by 2D ferroelectric heterostructure. (a) 3D schematic of the device, showing light coupled from an optical fiber via edge coupling into the waveguide integrated with the proposed 2D heterostructure under applied electrical pulses. (b) Cross‐section of the stacked 2D heterostructure (WS_2_/CIPS/Gr) on top of the SiN waveguide. (c) Simulated mode profile of the bare SiN waveguide without 2D material. (d) Simulated mode profile with the proposed heterostructure, highlighting enhanced light–matter interaction due to the higher refractive index of CIPS compared to SiN, enabling large phase modulation. (e) Numerically calculated phase shift in the waveguide arising from the index change in WS_2_ under electrical doping. The inset shows an optical image of the fabricated device. (f) Experimentally measured transmission response confirming the phase shift.

To realize a practical non‐volatile photonic phase shifter, we transferred the WS_2_/CIPS/Gr heterostructure on a SiN microring resonator (MRR), an essential component in PICs due to its high sensitivity to any index change and compact design. The SiN waveguide is engineered for single‐mode propagation at 1550 nm, with a thickness of 350 nm and a width of 1.2 µm. The MRR has a radius of 100 µm, yielding a free spectral range (FSR) of 1.8 nm and a loaded Q of approximately 35000 (Figure S1). The heterostructure is transferred precisely over the ring, with Gr and WS_2_ serving as transparent, electrostatically gated electrodes and index change materials. Light is edge‐coupled into the waveguide using a tapered optical fiber, as shown in Figure 3a, the inset also shows the optical microscope image of fabricated heterostructure over the MRR, with an active overlap of 15 µm. The cross‐sectional view (Figure 3b) displays the vertical stacking of monolayer WS_2_, 50 nm thick CIPS and monolayer Gr on top of the SiN waveguide. The WS_2_ and Gr are contacted with the Ti/Au (10/40 nm) pads. Using the electrical probe, electrical pulses from a pulse generator with different voltages and duty cycles are applied to study the corresponding transmission at the other end of the device.

The optical modulation or index change arises primarily from the electrostatic doping induced in the WS_2_/graphene layers by the polarized Cu^+^ ions. CIPS alone produces only minimal refractive‐index modulation, and the strong phase‐tuning behavior results from the polarization‐driven charge redistribution in the adjacent 2D layers. Numerical simulations were performed for both the bare and the heterostructure‐loaded waveguide geometries. Importantly, the refractive index values used in the model were extracted from DFT calculations to match the experimental shifts observed in the transmission spectra. As shown in Figure 3c, the bare SiN waveguide supports a tightly confined optical mode. However, after introducing the high‐index CIPS layer (*n* ≈ 4), the optical field is significantly pulled into the 2D heterostructure (Figure 3d), enhancing light–matter interaction and enabling a large effective index modulation. The index changes in WS_2_ under Cu⁺‐induced doping led to a substantial phase shift, as confirmed by numerical calculation (Figure 3e) and experimental transmission measurements (Figure 3f). From the recorded spectrum and resonance shift, we have calculated the change in effective refractive index. Compared to previous works [27], the achieved phase shift is notably larger, enabled by the combined effects of ferroelectric domain switching and the strong confinement in the high‐index CIPS layer.

**FIGURE 4:**
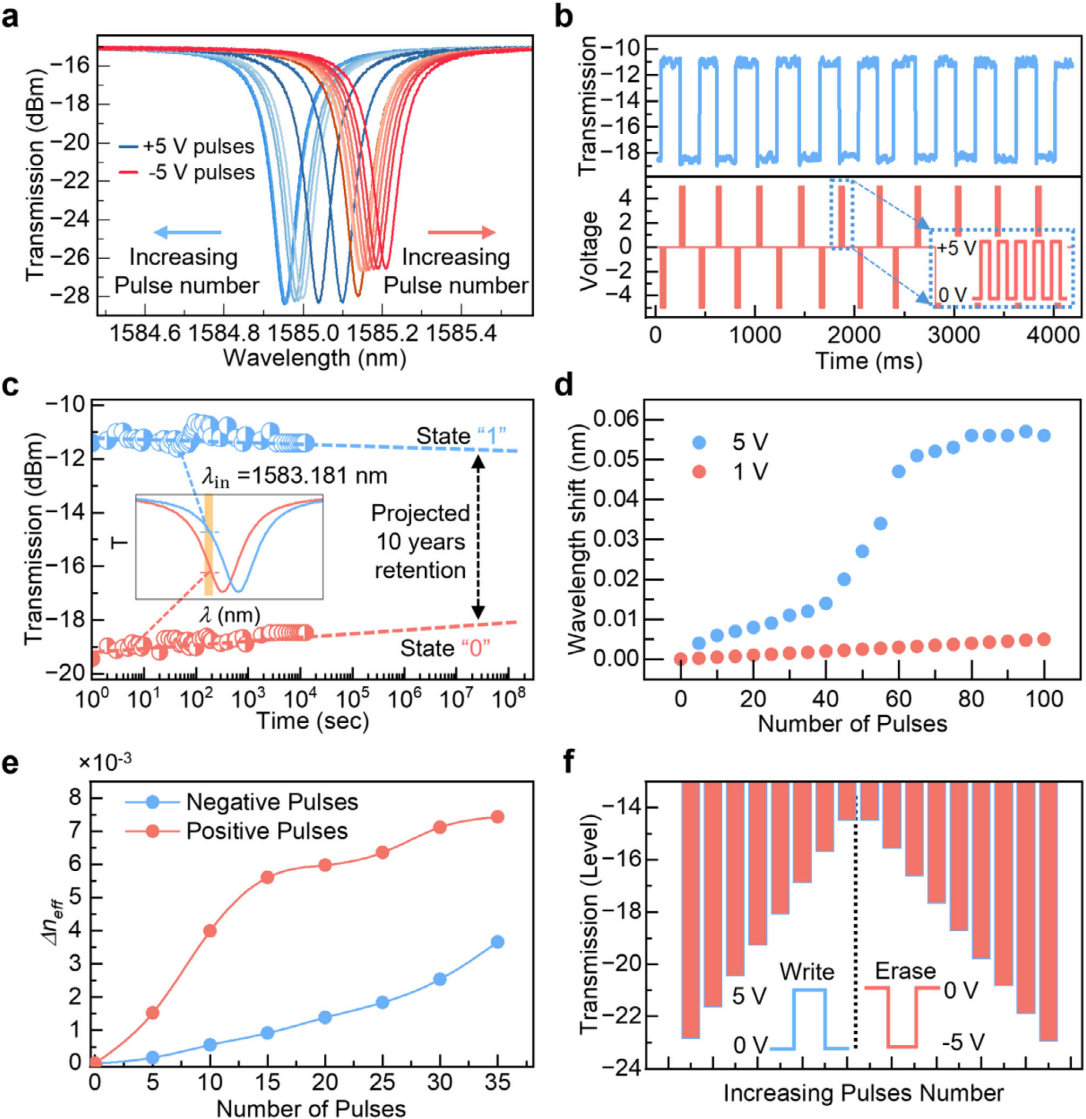
Demonstration of electrically programmed and retained resonance shifts. (a) Resonance wavelength shifts observed under the application of multiple +5 V (blue, blueshift) and ‐5 V (red, redshift) pulses, starting from an initially unpolarized (random domain) state. (b) Applied electrical pulse sequence (bottom) and corresponding modulation of optical transmission intensity (top) at a fixed resonance wavelength. (c) Retention characteristics demonstrating bistable optical states over an extended duration, indicating non‐volatile behavior. (d) Dependence of resonance wavelength shift on the amplitude of the applied voltage pulses. (e) Effective refractive index change (Δ*n*_eff_) as a function of applied electric field. (f) Stepwise wavelength shift showing an initial blueshift followed by a return to the center wavelength and then a redshift, illustrating reversible domain switching.

To experimentally demonstrate non‐volatile phase modulation, we applied controlled electrical pulses to switch the ferroelectric domains in CIPS and monitored the resulting transmission spectral changes (Figure 4a). Starting from a randomly polarized state, we first applied a sequence of 5 V, 50 ms positive pulses to align the domains in one direction. Subsequently, short 5 V, 1 ms positive pulses induced a progressive blueshift in the resonance wavelength, as shown in Figure 4a. Reversing the polarity to ‐5 V triggered a redshift, indicating domain switching in the opposite direction and confirming the bistable and reversible nature of the modulation. When the full polarization sweep is considered, the total non‐volatile resonance shift reaches approximately 0.15 nm. A sequence of alternating positive and negative pulses returned the domain configuration to a randomized state. Further application of long ‐5 V, 50 ms pulses fully aligned the domains in the opposite direction, resulting in increased optical absorption and a measurable decrease in extinction ratio (∼2 dB) at the resonance dips, which is consistent with the DFT‐predicted increase in Gr absorption.

To capture the time‐dependent transmission response, we monitored the output intensity at a fixed wavelength (near to resonant wavelength) while applying bursts of electrical pulses. A series of five positive pulses (5 V, 5 ms) was used to reorient the ferroelectric domains in CIPS, resulting in a sharp increase in transmission due to the induced phase shift (Figure 4b). This intensity rise corresponds to a blueshift in the resonance, caused by the Cu^+^ ions aligning away from the WS_2_ side and reducing its optical absorption. When the polarity of the pulses is reversed (‐5 V), the domains switch in the opposite direction, leading to a redshift and a corresponding decrease in transmission. To enhance the contrast in transmission and ensure full switching of the domains, we used a train of five pulses for each polarity cycle.

We fabricated and tested three independent devices and observed a similar tuning range, confirming that the non‐volatile wavelength detuning is repeatable across multiple repetitions and devices. As for practical applications, the relevant parameter is the intensity contrast obtained at a fixed wavelength, the phase information is typically converted into intensity for readout. Figure 4c demonstrates the non‐volatile nature of the phase modulation. In this experiment, the transmission was continuously monitored at a single resonance wavelength of 1583.181 nm over 2 × 10^3^ s and later discretely recorded after each 900 s up to 10^4^ s. Initially, the signal remained stable in the low‐transmission state “State 0.” After that, a burst of five positive 5 V, 5 ms pulses was applied, which switched the domains and shifted the resonance, causing a clear jump to a high‐transmission state, “State 1.” No further bias was applied after the switching event, yet the elevated transmission level remained stable for 10^4^ s, indicating strong non‐volatility and retention. This stable optical behavior, with sharp and persistent transitions between high and low states, confirms the ferroelectric origin of the modulation. Based on extrapolation from these measurements, the linearly projected data retention time exceeds 10 years, establishing the viability of this approach for in‐memory photonic computing. We also studied the dependence of modulation on pulse strength and pulse duration on the amount of the resonance shift. We measured the separation between these intensity levels and found that eight levels are clearly distinguishable with sufficient margin, which justifies our claim of eight‐level stable operation. Increasing the number of levels reduces the spacing between adjacent phase states, making them difficult to resolve reliably with the present device Q‐factor.

As shown in Figure 1, 4 msec 5 V induces larger resonance shifts compared to applied 1 msec pulse of 1 V. As we keep increasing the number of the pulses, we have found that after certain number of pulses the amount of shift little, largely because now domain orientation of all the vdW layers of CIPS is in one direction. Further increasing the pulse number results no shift as all the domains are completely aligned with the direction of applied voltage. The wavelength detuning shown in Figure 4d corresponds only to the incremental shift generated by applying programming pulses in a positive polarity only; when the full polarization changed the resonance shift gets as large as 0.15 nm. The effect of pulse width is also being studied and shown in Figure S8, along with the resonance shift from the second device Figure S8e. From the shift amount, the corresponding change in effective refractive index (Figure 4e) is calculated using the equations mentioned in Section S6. The change in the effective refractive index from the positive pulses is quite large from the random zero state of the device as WS_2_ contributs large change. The approximate estimation of polarization‐induced Index change and number of charge carrier change is calculated using the mode overlap calculated from the simulation, number of carrier changes due to bandgap change of both WS_2_ and Gr with respect to the position of the Cu^+^ position in the CIPS, in Section S4, shows the calculation and corresponding DFT calculated data is shown in Figure S2. To show the multilevel retention operation, a burst of five pulses of 5 msec is applied at a fixed resonance wavelength of 1581.18 nm and the transmission spectrum is recorded, (Figure 4f) shows the resonance wavelength initially shifting to the blue with positive pulses hence increasing intensity from the minimum resonance transmission, and it started redshifting under negative bias, clearly illustrating reversible and multi‐level stable phase tuning via ferroelectric domain control. We tested three independent devices and observed consistent non‐volatile phase‐modulation behaviour across all of them, confirming reproducibility. Each device was subjected to more than 2000 programming pulses, during which the modulation performance remained stable; degradation occurred only at the graphene and Au contact edge due to current crowding, not in the CIPS and heterostructure itself. This issue can be mitigated in future designs by optimizing the contact geometry to reduce local heating.

To benchmark the performance of our Gr/CIPS/WS_2_‐based phase shifter, we compare it against various state‐of‐the‐art modulation platforms in terms of switching energy, retention time, insertion loss, and active device length. As summarized in Table 1, our device demonstrates a unique combination of low switching energy (2.5 pJ), long retention time (> 10 years), and low insertion loss (∼0.2 dB)—surpassing most other platforms, including phase‐change materials, charge‐trapping, and bulk ferroelectrics. Unlike conventional 2D material‐based modulators, which typically suffer from volatility and limited retention, the integration of out‐of‐plane ferroelectric CIPS with a tunable WS_2_ enables non‐volatile, energy‐efficient phase modulation in a compact MRR configuration. This superior performance makes our device highly suitable for in‐memory photonic computing and ONN applications.

**TABLE 1:** Comparison of various non‐volatile programmable memory/modulator in on chip Silicon photonics.

Device mechanism	Switching Energy	Retention time	Insertion loss/absorption	Active device length	Mechanism (MRR/MZI/ other)
PCM [11, 23]	180 pJ–20 nJ	10 years	∼0.3 dB	∼15 µm	MRR/MZI
Charge‐Trapping [40, 41]	∼ 15 pJ	Few Years	∼1 dB	∼1 mm	MRR
Memristor [24, 42]	10–40 nJ	10 Years	∼1 dB	∼10–20 µm	Other
ferroelectric material (BTO etc.) [20, 21]	4.6–30 pJ	10 Years	∼0.05 dB	∼1 mm	MRR/MZI
Gr/CIPS/WS_2_ (this work)	2.5 pJ	> 10 years	∼0.2 dB	15 µm	MRR

Recently some researchers have shown optical convolution, and on‐chip matrix multiplication using MRR as programmable weight banks for ONN [1, 10, 11]. The stable and reversible non‐volatile switching behavior demonstrated by our device inspired its use in in‐memory photonic computing, particularly for ONN. Here, we exploit the ability to retain optical phase states to store and modulate the weights for a neural network directly in the photonic domain.

### 2.3 Photonic Neural Network Based on WS_2_/CIPS/Gr Nonvolatile Modulators

**FIGURE 5:**
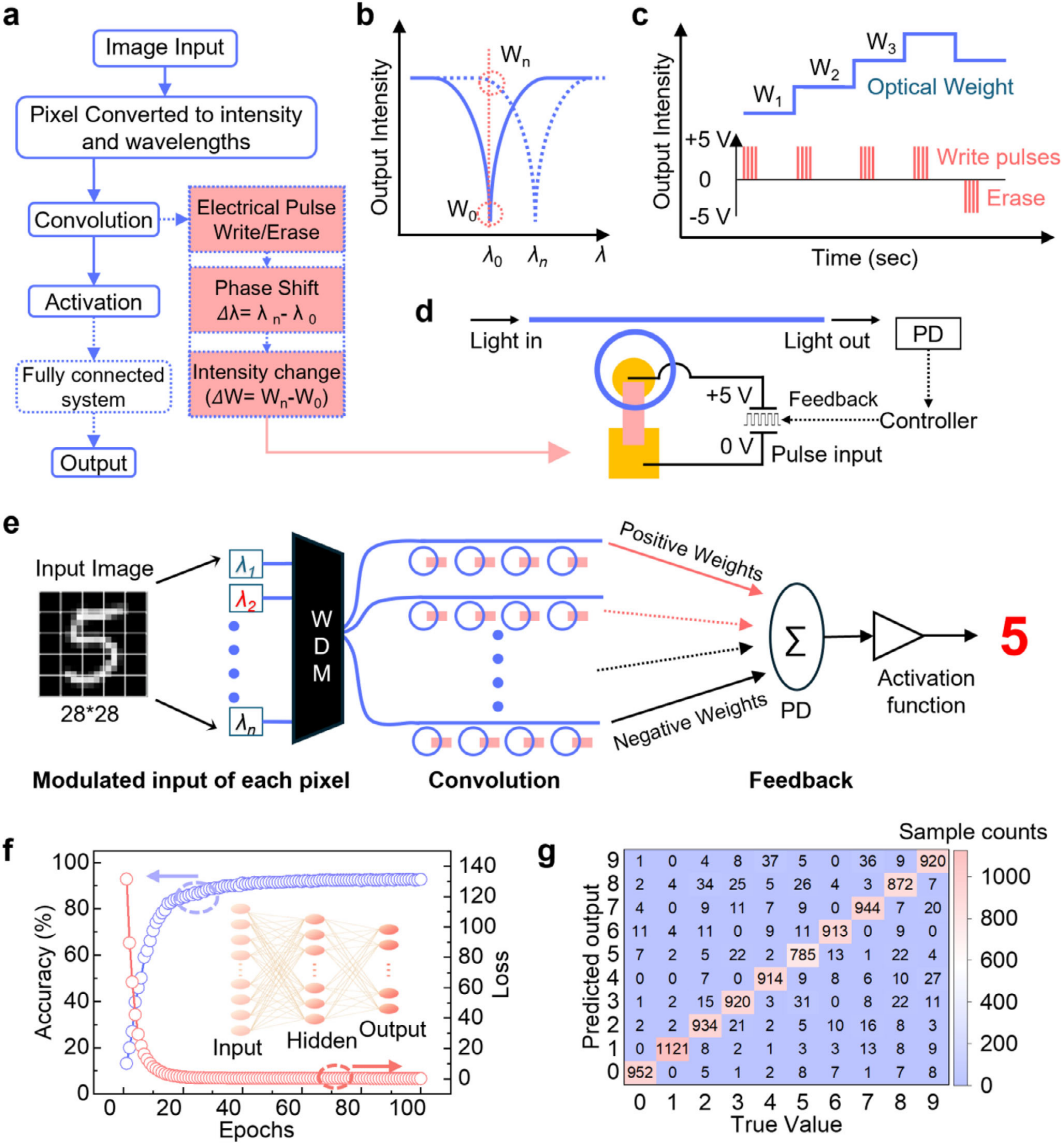
ONN simulation with programmable photonic synapses. (a) Block diagram illustrating the photonic neural network where input digits are encoded into wavelength channels, convolved via MRR‐based weights, and classified through Rectified linear unit (ReLU) activated layers. (b) Weight modulation through phase shifts in microring resonators, reflected as intensity variations in the transmission spectrum. (c) Input electrical pulses and the corresponding change in optical weights/intensity. (d) Schematic of a single‐ring unit, indicating the application of electrical pulses, an integrated photodetector, and a controller for system operation. (e) Schematic illustration of image edge detection using the proposed device, where convolution with a ReLU activation function enables number recognition with different wavelengths as inputs and the non‐volatile weights provided by the device. (f) Training results showing accuracy and loss versus epochs. (g) Example of classification performance: predicted versus true labels in a sample confusion table.

To demonstrate this concept, we used the MNIST handwritten digit dataset, which contains grayscale images of numbers 0–9. The results in Figure 5 are based on simulations, and the illustrated architecture is not yet physically implemented in our current prototype. Our aim here is not to claim a realized photonic processor, but to show how the experimentally demonstrated non‐volatile phase modulator can enable future system‐level applications. The simulations are grounded in experiment: we used the measured phase‐tuning information, converted into intensity, as weights from our fabricated MRR to train and evaluate the model, ensuring that the results reflect realistic device performance. As illustrated in Figure 5a, handwritten digits are first encoded into optical intensity, and each vector is mapped to wavelength channels, providing a parallelized representation of the input image. These optical signals are then processed through a weight matrix implemented by MRR with non‐volatile phase modulation, where each vector convolution is mapped onto a distinct wavelength channel. The resulting convolved outputs are passed through an activation stage, which introduces nonlinearity into the model. The effective weights of the network are subsequently updated during backpropagation based on the computed loss. By cascading and training multiple such arrays, the system can learn to recognize and classify the handwritten input with high accuracy, demonstrating the feasibility of non‐volatile photonic hardware for neural network inference.

To evaluate the functionality of the experimentally obtained discrete phase states in photonic neural networks, we implemented a simulation framework incorporating quantized synaptic weights. The device‐generated weight table *W* ∈ *R^L^*, with *L* discrete optical states extracted from transmission levels, served as the constraint for synaptic weights in the model. To enable differentiable training, we adopted the Gumbel–Softmax reparameterization [43, 44, 45], where the relaxed categorical distribution is given by:

(2) $$y=\mathrm{softmax}\left(\frac{z+g\;}{\tau }\right)$$

Here, *g* is sampled Gumbel noise, and *𝛕* is a temperature parameter controlling exploration. The effective weight becomes: $\omega_{eff}=\sum_{j=1}^{L}y_{j}W_{j}$ This formulation allows gradient‐based backpropagation through the discrete state space, mimicking device‐constrained optical weights. The network architecture comprises two fully connected multi‐wavelength linear layers: 784→128→10, with ReLU activation applied as: $f\;\left(x\right)=\;max\left(0,x\right)$. Training was conducted using the Adam optimizer and a cross‐entropy loss function, expressed as:

(3) $$L=-\frac{1}{N}\sum_{n=1}^{N}\log{\hat{p}}_{y^{\left(n\right)}}^{\left(n\right)}$$

where the predicted probability of class i for sample n is computed as:

(4) $${\hat{p}}_{i}^{\left(n\right)}=\frac{\exp\left(z_{i}^{\left(n\right)}\right)}{\sum_{j=1}^{C}\exp\left(z_{j}^{\left(n\right)}\right)}\;,i=1,\cdots \cdots ,C$$

Here, *z*_i_^(n)^ denotes the unnormalized logit, *y^(n)^* is the true label, and *C* is the total number of classes. Step‐decay learning rate scheduling is applied to ensure convergence. This framework enables end‐to‐end training while embedding real device constraints, validating the practicality of the non‐volatile, discrete phase states in photonic in‐memory computing, and achieving 92% accuracy on the MNIST handwritten digit dataset.

The mechanism of weight programming is further illustrated in Figure 5b, where optical phase shifts are achieved by applying short electrical pulses that alter the ferroelectric domain state in CIPS. The resulting shift in phase corresponds to the intensity change at a given fixed wavelength. The resulting change in refractive index within the WS_2_ layer modulates the effective phase of the optical signal. The dynamic relationship between pulse input and optical weight response is shown in Figure 5c, highlighting how multiple discrete levels can be programmed through successive voltage pulses. This multi‐level control is essential for in‐memory computing. Our device demonstrates clear advantages in low loss, minimal energy consumption, and endurance, enabled by the ferroelectric switching mechanism. The complete layout of a single photonic synaptic unit is shown in Figure 5d, including the integrated electrodes, photodetector, and control logic needed for system‐level operation and electrical programming.

To further explain how the full system looks like, in Figure 5e, the input optical image is flattened into a set of vectors at an optical wavelength, where each pixel value is encoded into optical intensity. These optical signals are then multiplexed onto different wavelength channels and launched simultaneously into the system, providing a parallelized optical representation of the input. This can be done using wavelength division multiplexing (WDM), where multiple wavelengths are encoded together into a single waveguide. The encoded light is then parallelly routed through an array of MRRs, each functioning as a programmable weight. These MRRs are configured with our non‐volatile 2D heterostructure‐based phase shifters, which set the phase weights based on prior electrical programming. Since all MRRs operate in parallel, this enables fast and energy‐efficient convolution, greatly reducing the time required compared to serial electronic processing. In the ONN simulation, we used a ReLU activation function and performed the convolution operation optically by multiplying the input pixel intensities (encoded as wavelengths) with the stored phase weights in the rings. We used this architecture to simulate a single layer to classify handwritten digits from the MNIST dataset. Optical weights were programmed onto the MRR, and wavelength‐encoded image data was passed through the photonic circuit for inference. Figure 5f shows the training accuracy and loss over epochs, achieving 92% recognition accuracy with a ReLU‐based activation model. Finally, Figure 5g presents a confusion matrix comparing predicted versus true labels, confirming high classification fidelity across most digit classes and validating the effectiveness of our non‐volatile photonic synapses in neural inference tasks. Together, these results highlight the promise of integrating 2D ferroelectric heterostructures with photonic circuits to realize programmable, non‐volatile ONNs. Such devices offer a pathway to scalable, energy‐efficient, and in‐memory optical computing, with direct implications for future on‐chip AI accelerators.

## 3 Conclusion

Our work demonstrated non‐volatile phase modulation using a vdW heterostructure that integrates monolayer WS_2_ and Gr with the dielectric ferroelectric CIPS. Leveraging ferroelectric domain switching in CIPS and the resulting electrostatic doping‐induced index modulation in WS_2_, the device achieves a large, stable, and reversible phase shift with low optical loss, multilevel operation up to 8‐bit, and an energy consumption of only 2.5 pJ per programming cycle. Owing to its capacitive nature, the device consumes zero static power, requiring only short voltage pulses to set and retain the phase state. The out‐of‐plane ferroelectric polarization in CIPS ensures long retention and high endurance, establishing robust non‐volatile performance. Unlike conventional volatile phase modulators that rely on continuous biasing, our device combines non‐volatility, fine phase resolution, and low‐loss operation within a compact integrated platform. We further demonstrate that the programmable phase states can act as optical weights for photonic circuits, directly enabling scalable ONN and photonic computing architectures. These results highlight our proposed 2D ferroelectric heterostructures as a route to energy‐efficient, low‐loss, and non‐volatile photonic systems, paving the way for neuromorphic and large‐scale reconfigurable optical technologies.

## 4 Experimental Section

### 4.1 SiN Waveguide Fabrication

A 350 nm thick SiN layer was deposited on a thermally oxidized SiO_2_ substrate using low‐pressure chemical vapor deposition. The waveguides were then defined in AR‐P 6200 e‐beam resist by electron‐beam lithography (Raith EPBG, 100 kV). Following resist development, the patterns were transferred into the SiN using inductively coupled plasma reactive ion etching with CHF_4_ as the primary etchant, while Ar was co‐flowed to regulate etch rate and chamber pressure. Residual resist was removed by oxygen plasma ashing. The passive Si_3_N_4_ structures were clad with ∼1 µm of SiO_2_ deposited by plasma‐enhanced chemical vapor deposition, and the top surface was subsequently planarized by chemical mechanical polishing, leaving a residual SiO_2_ layer of 50–100 nm and achieving a surface roughness below 1 nm. For electrode formation, the sample was spin‐coated with a positive photoresist (AZ5214E) and patterned using a direct laser writer (DML 3 Pro). Ti/Au (10 nm/40 nm) was deposited by thermal evaporation, and lift‐off yielded the final metal contacts, as illustrated in Figure 3a. A final oxygen plasma treatment rendered the surface hydrophilic, facilitating vdW integration of 2D flakes. The active waveguide region was aligned adjacent to the electrodes, and the thickness of transferred flakes was verified by an atomic force microscope.

### 4.2 Non‐Volatile Transmission Spectra Measurement

To characterize the optical response and phase shift behavior of the device, we performed wavelength‐resolved transmission measurements using a Santec TSL‐510 tunable laser, sweeping across the 1510–1600 nm wavelength range. The output from the tunable laser was routed through a polarization controller via single‐mode fiber to ensure transverse electric polarized light injection. The controlled polarization state was then coupled into the device under test using a tapered lensed fiber, aligned to the edge‐coupled SiN waveguide. The transmitted light from the output facet of the waveguide was collected using another lensed fiber and detected using a high dynamic range logarithmic photodetector. The detector was connected to a time‐synchronized data acquisition system, allowing real‐time tracking of the transmission spectra. All measurements were controlled via a custom PC interface that triggered the laser sweep and collected the data. To apply electrical programming pulses, a Keysight pulse generator was used in conjunction with high‐precision electrical probes, aligned onto the Au contact pads of the Gr and WS_2_ electrodes. The pulse amplitude, width, and frequency were precisely controlled, and for each pulse condition, a complete wavelength sweep was conducted. The resulting spectra were recorded after each electrical state change to analyze the resonance shifts and extinction ratio variations due to domain switching.

### 4.3 The Device Fabrication and Characterization

The WS_2_/CIPS/graphene heterostructure was assembled using a deterministic dry‐transfer process based on a polycarbonate (PC) stamp mounted on a transparent glass slide. Monolayer WS_2_, few‐layer CIPS, and monolayer graphene were first mechanically exfoliated onto SiO_2_/Si substrates using standard Scotch‐tape exfoliation. Suitable flakes were identified under an optical microscope by their color contrast and lateral dimensions, and their thicknesses were verified using atomic force microscopy (AFM). As shown in Figures S6 and S7, each selected flake was sequentially picked up by gently lowering the PC stamp onto the substrate and adjusting the temperature to achieve adhesion. The stacked PC/heterostructure was then aligned with the pre‐patterned SiN microring resonator and gold contacts, and the entire stack was released by heating the substrate to 180°C. Finally, the sample was immersed in chloroform for 12 h to dissolve the PC layer, resulting in a clean and well‐aligned heterostructure accurately positioned over the desired waveguide region.

### 4.4 FDTD Simulation

To quantify the impact of refractive index changes in WS_2_ on optical phase modulation, we first performed mode simulations to calculate the effective index change in the SiN waveguide upon integration with the WS_2_/CIPS/Gr heterostructure. By varying the refractive index of WS_2_ based on values obtained from calculations, we determined how light confinement and phase propagation in the waveguide were affected. Subsequently, a full MRR simulation was carried out using Lumerical FDTD. The geometry included an MRR coupled to a bus waveguide, with material properties assigned to match experimental parameters. The simulation space was enclosed by perfectly matched layers to eliminate boundary reflections. A broadband optical source was launched into the bus waveguide to excite resonant modes, and field monitors were placed at strategic points to record electromagnetic fields. To ensure numerical accuracy, particularly near the ring‐waveguide coupling region and heterostructure interface, a refined non‐uniform mesh was applied. The collected time‐domain data were transformed into the frequency domain using Fourier analysis to extract the transmission spectrum, including key parameters such as resonance wavelengths, Q‐factor, and free spectral range (FSR). By correlating the simulated effective index shift with experimentally observed resonance shifts, we confirmed the modulation behavior and estimated the phase shift efficiency of the device.

## Funding

Singapore Ministry of Education (MOE) Academic Research Fund (AcRF) Tier 1 grants (RG63/23, RT2/23, RG71/25) and Tier 2 (MOE‐T2EP50224‐0018), MOE AcRF Tier 3 grant (MOE‐MOET32023‐0003), Individual Research Grant (IRG) under the Project M23M6c0109, ASTAR IRG (M23M6c0102)

## Conflicts of Interest

The authors declare no conflict of interest.

## Supporting information




**Supporting File**: advs73440‐sup‐0001‐SuppMat.docx.

## Data Availability

The data that support the findings of this study are available from the corresponding author upon reasonable request.
