# Supplementary material for: Non‐Volatile Phase Modulation with Ultralow Energy Consumption Enabled by 2D Ferroelectric/TMD Heterostructures
Source: Adv Sci (Weinh). 2026 Jan 28;13(13):e20795. doi: 10.1002/advs.202520795 (PMC12955936; doi:10.1002/advs.202520795)
Supplement: Supplementary file 1 — Supporting File: advs73440‐sup‐0001‐SuppMat.docx. [file ADVS-13-e20795-s001.docx]

**Supporting Information for**

**Non-volatile phase modulation with ultralow energy consumption enabled by 2D ferroelectric/TMD heterostructures**

*Lalit Singh^1^*, Shi Guo^1^*, Yuhui Yang^1^*, Sholehin Juperi^1,2^, Rui Yu^1^, Xiangxin Gong^1^, Jeremy Leong^1,2^, Sung-Gyu Lee^1,2^, Qingyun Wu^3^, Lay Kee Ang^3^, Sang Hoon Chae^1,2,4^*^✉^

^1^School of Electrical and Electronic Engineering, Nanyang Technological University, Singapore, 639798, Singapore.

^2^CNRS-International-NTU-Thales Research Alliance (CINTRA), IRL 3288, 50 Nanyang Drive, Singapore, 637553, Singapore.

^3^Science, Mathematics and Technology, Singapore University of Technology and Design (SUTD), 8 Somapah Road, Singapore 487372, Singapore.

^4^School of Materials Science and Engineering, Nanyang Technological University, Singapore, 639798, Singapore.

* These authors contributed equally.

E-mail: sanghoon.chae@ntu.edu.sg

**Table of Contents**

1. **Propagation loss and Insertion losses calculation**
2. **Ideal power consumption and Switching energy calculations**
3. **DFT Calculations**
4. **Estimation of index change from the band bending calculation**
5. **Broad band spectrum**
6. **The model to calculate the** ***Δn_eff_* for the microring resonator**
7. **Fabricated device image**
8. **Results from second device and pulses variation effect**
9. **AFM images and Thickness calculations**
10. **Raman spectroscopy**
11. **PFM measurement**
12. **Propagation loss and Insertion losses calculation**

To quantify propagation loss in the microring resonator waveguides, we measured transmission spectra from multiple nominally identical devices located across the chip. Using the procedure of Ref. 1, we first separated coupling loss and then inferred the propagation loss. The loss coefficient ⍺ and coupling coefficient *t* were obtained by:

$$(⍺,t)=\left( \frac{A}{B} \right)^{\frac{1}{2}}\pm\left( \frac{A}{B}- A \right)^{\frac{1}{2}}$$

where A and B are functions of the finesse, *F*, and extinction ratio, *r_E_,* of the resonance:

$A=\frac{cos\left( \frac{\pi}{F} \right)}{1+sin\left( \frac{\pi}{F} \right)}$ and $B=1 -\left( \frac{1-cos\left( \frac{\pi}{F} \right)}{1+cos\left( \frac{\pi}{F} \right)} \right)\frac{1}{r_{E}}$

where *F* = FSR/FWHM, FSR is the free spectral range of the device, and FWHM is the full width at half maximum of the resonance. A measured spectrum is shown below in Figure S1 with the relevant extracted parameters.


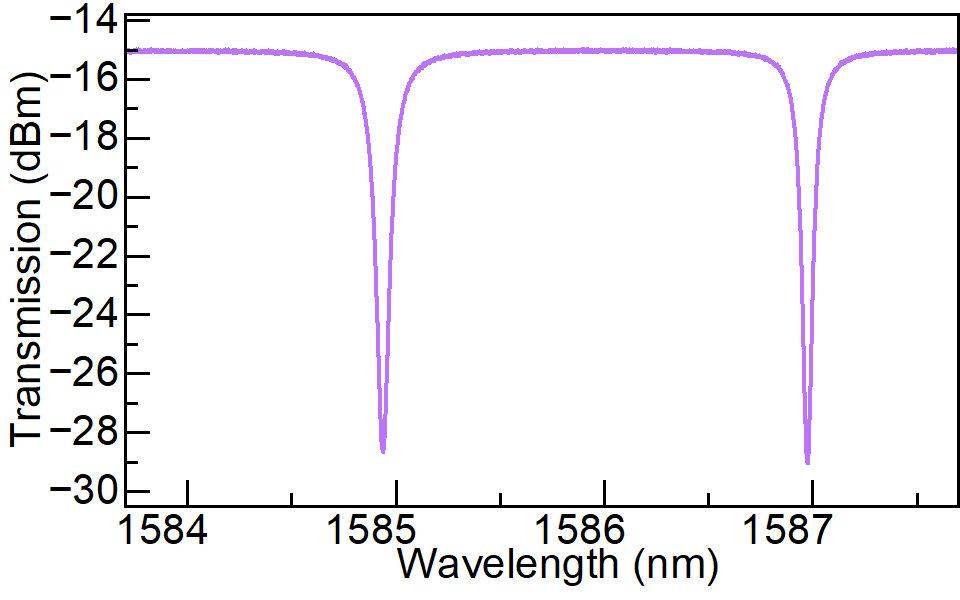


**Figure S1. For parameter extraction**. Resonant two peak of the optical transmission with ER= 14.2 dB, FSR of 2.1 nm and FWHM of 0.05 nm.

The loss coefficient α, is the optical loss in one roundtrip of the resonator. For the sample spectrum (Fig. S1) ⍺ = 0.953. From this we can calculate the propagation losses in dB/cm, using the circumference, L of the resonator. $p = \frac{10 {log}_{10}. \alpha}{L}$ Two devices were used to perform this analysis and extraction of the propagation losses. We extracted a mean propagation loss and standard error of 2.2 ± 0.2 dB/cm. The active region is defined by the overlap of the device on the waveguide, making the active region, length of the electrodes. Each device phase shifters is only *l* = 15~20 μm. Thus, the insertion loss is the propagation loss at full length ring 0.25 dB.

1. **Ideal power consumption and Switching energy calculations**

To calculate the ideal power withdrawn and Switching power, we used the following approach:

Power consumption while switching pulse input using $P = V \times I$:

We considered the situation with the highest extinction ration of the intensity:

- Pulse duration = 50 ms (but this affects energy, not average power unless averaged over time)
- Number of pulses = 5 (again affects energy if over time, not instantaneous power)
- Current I < 0.01 nA = 1 ✕ 10^-11^ A, Voltage = 5 V
- $P = V \times I$ Where: P is power in watts (W), V is the voltage (in volts), I is the current (in amperes) $P = 5 V\times1 \times{10}^{-11} A= 5 \times{10}^{-11}= 50 pW$
- To calculate energy withdrawn per pulse, use**: $E = P \times t = V \times I \times t$;** with 5 V, 0.01 nA, and 50 ms:

E= 5 $\times$10^-11^ W $\times$ 50$\times$10^-3^ s = 2.5 $\times$ 10^-12^ J = 2.5 pJ per pulse

In case of ideal: Because CIPS is an insulating dielectric (ε_r_ ≈ 15) with negligible DC leakage, essentially no current flows under a DC bias, yielding ~zero static power consumption.

Switching energy calculations using $E_{\text{sw}}\approx\frac{1}{2}CV^{2}$:

The CIPS dielectric exhibits $\varepsilon_{r}\approx7-10$ in the 1–100 kHz range.^1^ From this calculated capacitance of $C\approx279 \text{fF}$ and a input pulse of $V\approx1 V to 5 \text{V}$, the dynamic switching energy is approximated by $E_{\text{sw}}\approx\frac{1}{2}CV^{2}$ , yielding $E_{sw}\approx0.5\times279 fF\times\left( 1V to 5V \right)^{2}\approx0.2 pJ to 3.49pJ$ per transition. While DC leakage is extremely low (>0.001 nA). Consequently, leakage energy during typical pulse widths (1 ms–10 µs) is negligible: $E_{leak}\approx IVt\sim\left( 0.001 nA\times5 V \right)\times\left( {10}^{-3}-{10}^{-5}s \right)\approx50-0.05 fJ$ , which is <1% of the dynamic term.

1. **DFT Calculations of CIPS and effect on WS_2_ and Gr band bending**

First-principles calculations based on density functional theory (DFT) were carried out using Quantum ATK ^2^to examine the electronic properties of WS2/CIPS and Gr/CIPS. A k-point mesh of 5 × 5 × 1 was used for the geometry optimization and the property calculations, both based on the Monkhorst–Pack method.^3^ The atomic force criterion for the geometry optimization was set at 0.01eV/Å. The generalized gradient approximation (GGA) with the Perdew–Burke–Ernzerhof form was selected for the exchange-correlation functional.^4^ The DFT-D3 method with the Grimme scheme was adopted to account for the weak van der Waals interactions in the system.^5,6^ To eliminate interactions from periodic images, a 20 Å thick vacuum layer was inserted between adjacent images.


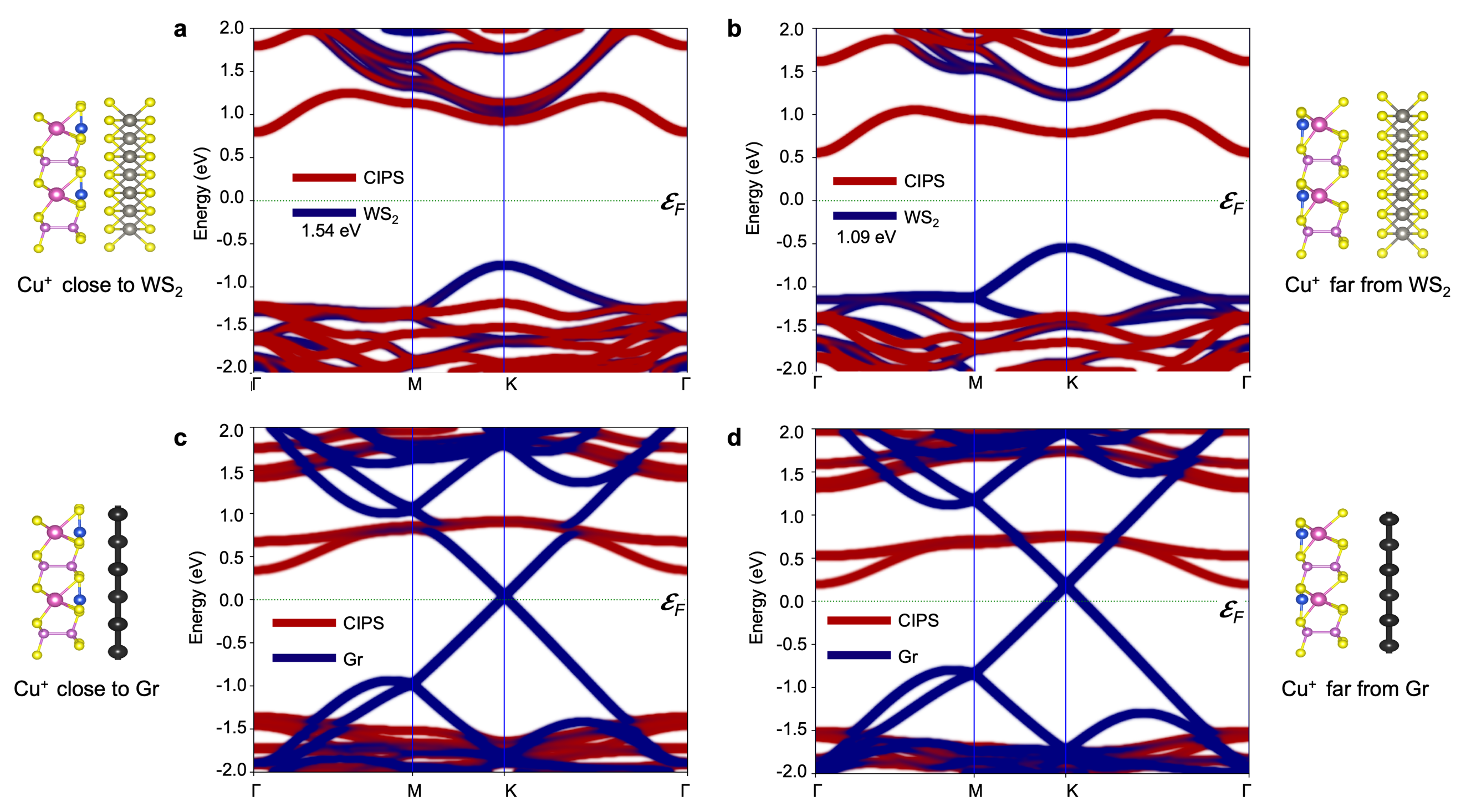


**Figure S2. DFT calculation for Bandgap analysis:** effect on the WS_2_/CIPS band gap when Copper ions are (a) near to the WS_2_ (1.54 eV) and (b) when away (1.09 eV). Effect on the Gr/CIPS band gap when Copper ions are (c) near and (d) when away.

1. **Estimation polarization-induced Index change**

**What drives your *Δn:* these contributions:**

1. Free-carrier (Drude/plasma) in WS₂ from polarization-induced doping
   - For a 2D sheet, use surface conductivity $\sigma_{s}\left( \omega\right)=\frac{n_{2D}e^{2}\tau}{m^{*}\left( 1-i\omega\tau\right)}$
   - Convert to a WS₂ permittivity change and then to waveguide $\Delta n_{\text{eff}} viaoverlap \Gamma:$


$$\Delta n_{\text{eff}}\approx\Gamma_{\text{WS2}}\frac{\Delta\varepsilon_{\text{WS2}}}{2n_{\text{WS2}}}$$

2. Band-edge (and exciton) shift from the gap change $E_{g}\to E_{g}-\Delta E_{g}$ modifies $n\left( \omega\right)$.

**Estimation polarization-induced carriers:**

1. Polarization charge from CIPS^7,8^: typical *P~* a few μC cm⁻²

Surface charge carrier density $n_{2D}\approx f P/e$ With $P\sim3 \mu\text{C }\text{cm}^{-2}\Rightarrow P/e\approx1.9\times{10}^{13} \text{cm}^{-2}$

f (0–1) based on screening split between graphene and WS_2_

1. Intrinsic carriers: $n_{i}\propto\sqrt{N_{c}N_{v}} e^{-E_{g}/\left( 2kT \right)}$
   1. From DFT calculation: E_g_ goes 1.54 → 1.09 eV (ΔE_g_ = 0.45 eV).
   2. At 300 K, 2kT $\approx0.052eV\Rightarrow e^{\Delta E_{g}/\left( 2kT \right)}\approx e^{0.36/0.052}\approx{10}^{3}$
   3. So n_i_ rises by ~10^3^.
2. From Moss relations^9^

$$\left( \frac{n_{2}}{n_{1}} \right)^{4}=\frac{E_{g1}}{E_{g2}}$$

3. Project to the ring$:$

$\Delta n_{\text{eff}}=\Gamma_{{WS}_{2}} .\Delta n_{{WS}_{2}}$

$\Delta\phi=2\pi L \Delta n_{\text{eff}}/\lambda$

1. **Broadband Spectrum**

We measured and normalized the broadband transmission spectrum of the microring resonator over 1510 nm -1600 nm using a Santec TSL-510 tunable laser and a high-dynamic-range logarithmic photodetector. The laser output was power-leveled, and the recorded trace was normalized to a reference waveguide to remove source and coupling variations, yielding an absolute baseline for comparison across the full band. The high dynamic range of the log detector cleanly resolves deep resonance notches and weak side features, enabling reliable extraction of key parameters such as free-spectral range, linewidth, and extinction across the entire sweep.

**
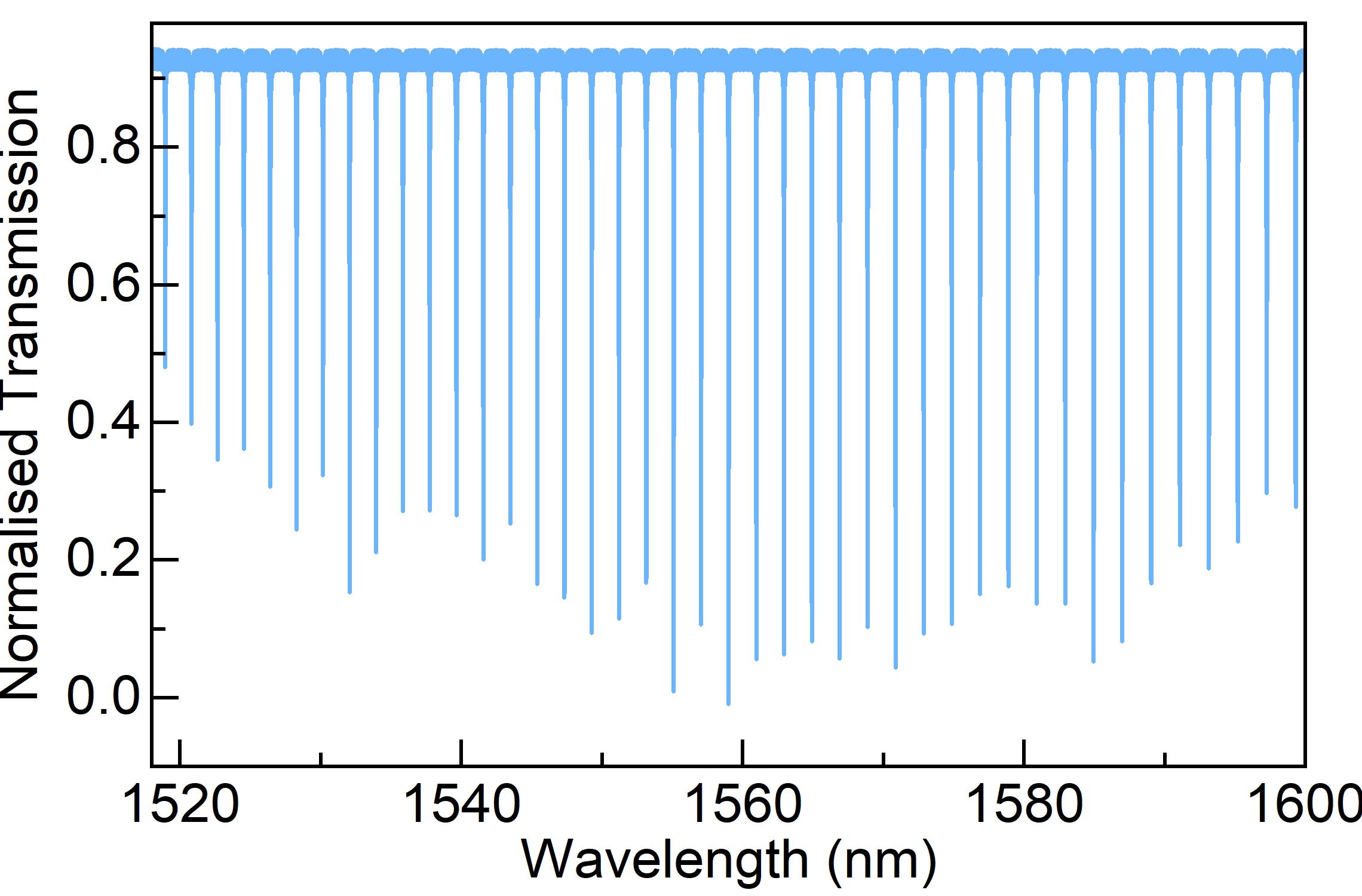
**

**Figure S3. Broadband Spectrum of microring resonator device**. Optical transmission as a function of wavelength.

1. **The Model to calculate the *Δn_eff_* for the microring resonator**

From the measured transmission spectrum after fitting we calculated basic parameters like FSR, FWHM, resonant peaks shift wavelength with respect to applied electrical pulses. To convert the bias-induced resonance shift into a modal index change, we used the standard ring-resonance relations.^10^ A resonance at the unbiased wavelength $\lambda_{v=0}$ shifts by $\Delta\lambda_{0}$ under bias the modal index change follows as:

$$\Delta n_{eff}=\frac{\Delta\lambda_{0} \lambda_{v=0}}{R FSR L}$$

Where L is the circumference of the micro ring resonator, R is the active coverage length of the heterostructure on the ring, FSR is the free spectral range.

1. **Fabrication steps and optical images and SEM images**


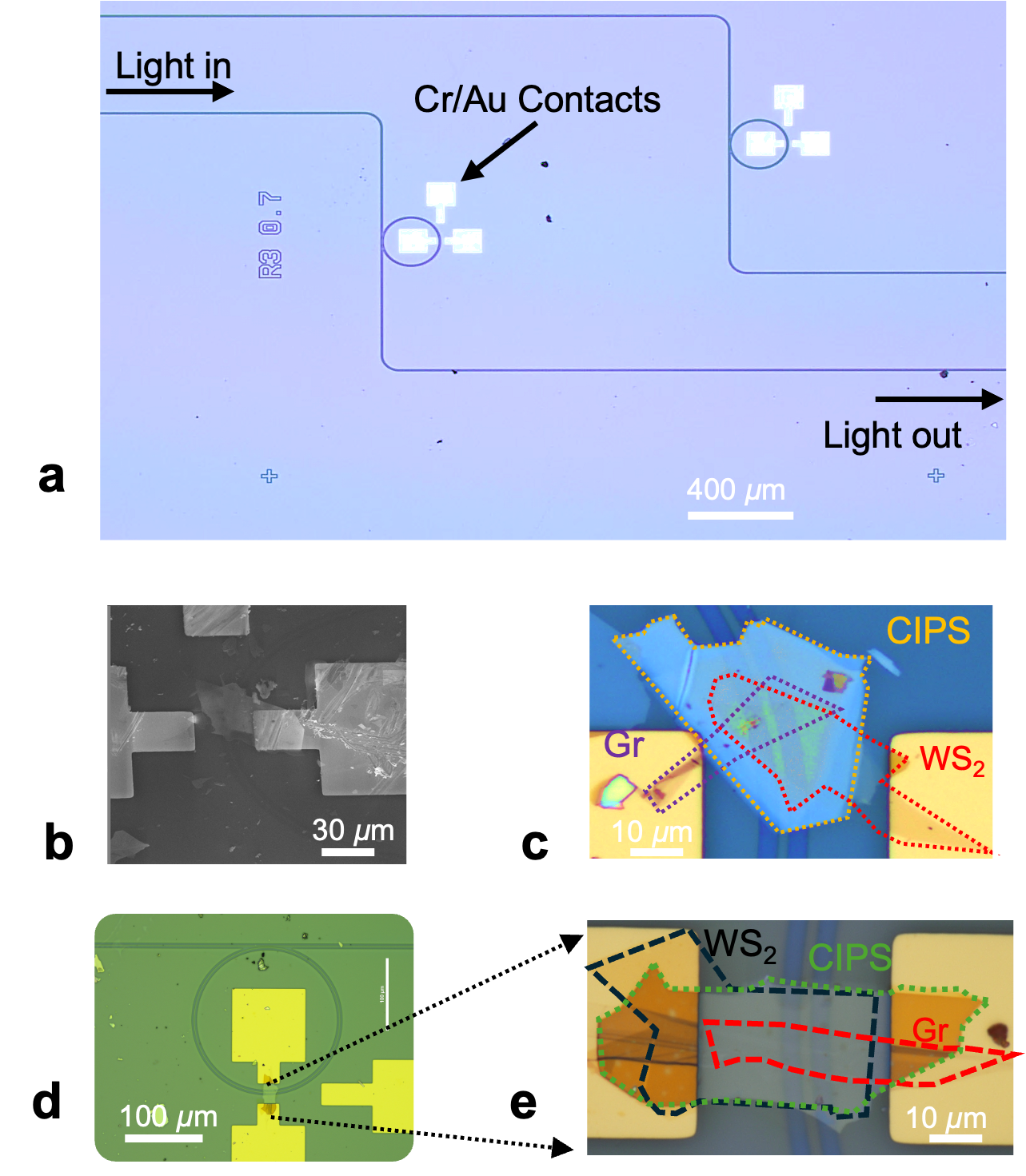


**Figure S4. Optical and SEM image of fabricated device** a) Full view of the fabricated chip showing SiN waveguides, edge couplers, metal contact pads, and integrated microring resonators. (b) SEM image of the heterostructure stack (WS_2_/CIPS/Gr) precisely aligned on the ring resonator of Device 1. (c) Optical microscope image of Device 1 showing the heterostructure placement over the ring. (d) Optical image of Device 2 with heterostructure integrated on a separate ring resonator. (e) Zoomed-in optical view of the heterostructure stack on the ring resonator of Device 2.

Figure S4 presents the optical and SEM characterization of the fabricated devices. The full-chip view shows the SiN waveguides, edge couplers, metal contact pads, and microring resonators used in our platform. SEM and optical images of Devices 1 and 2 confirm that the WS_2_/CIPS/graphene heterostructure is cleanly transferred and precisely aligned on top of the ring resonators. The zoomed-in views further verify accurate placement of the stacked 2D layers over the active region, ensuring effective interaction between the guided optical mode and the heterostructure.


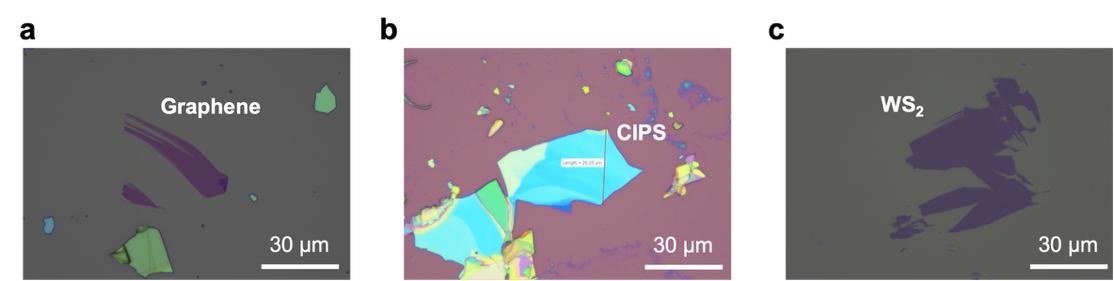


**Figure S5.** **To confirm clean heterostructure fabrication and transfer** (a) Graphene flake exfoliated onto a SiO_2_/Si substrate. (b) CIPS flake exfoliated on a SiO_2_/Si substrate. (c) WS_2_ flake exfoliated on a SiO_2_/Si substrate.


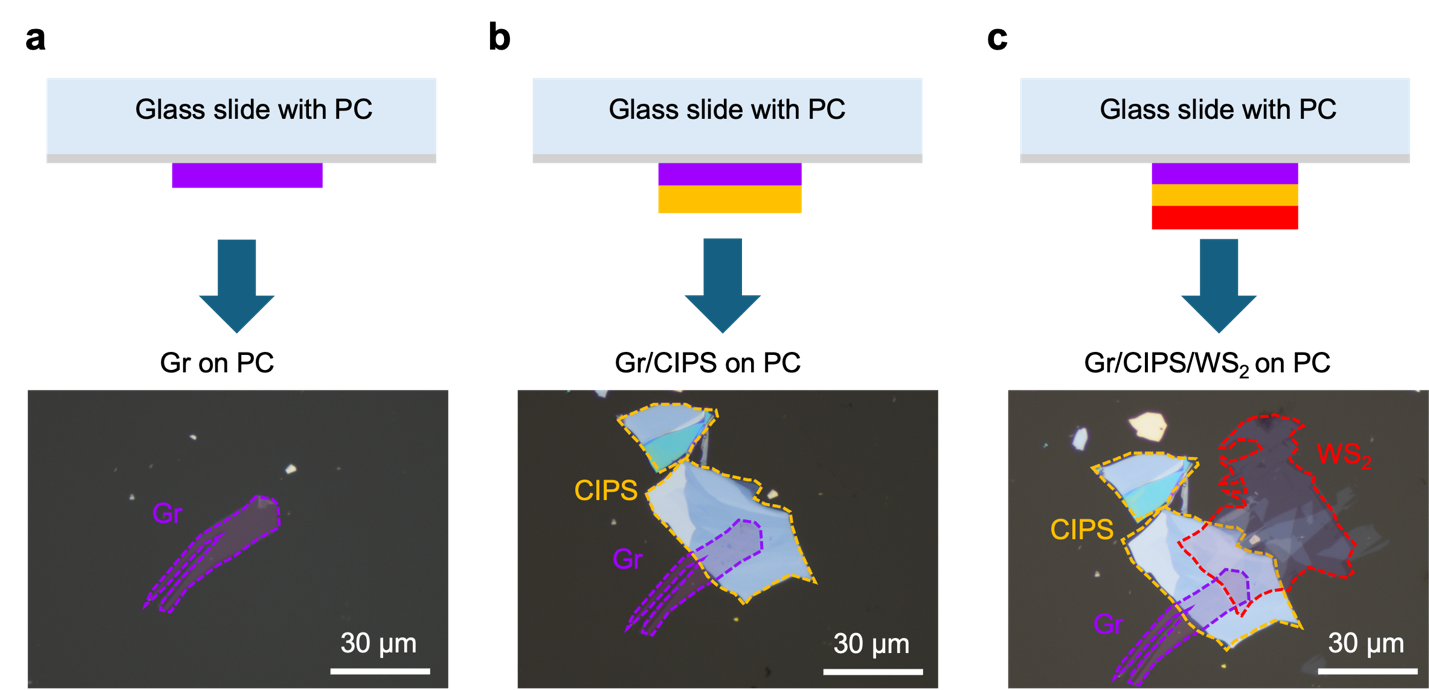


**Figure S6.** (a) Microscope image showing graphene picked up on the PC stamp, with the glass slide visible on top. (b) The CIPS flake being picked up by the same PC stamp using controlled temperature where graphene is already present. (c) Final step where the WS_2_ flake is picked up to complete the heterostructure stack.


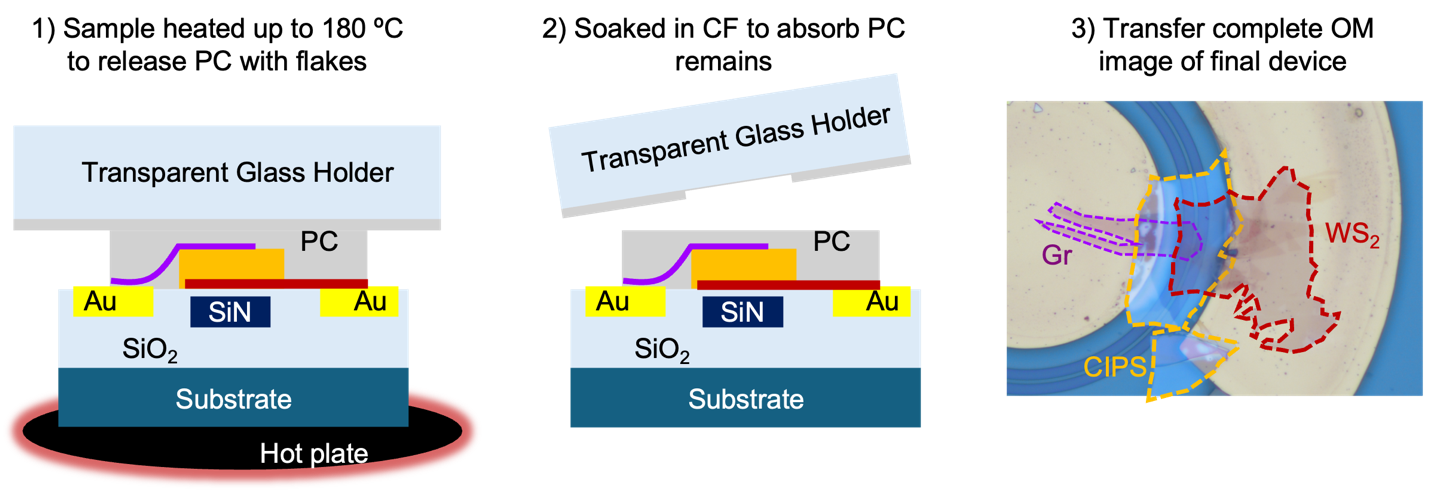


**Figure S7.** The complete WS_2_/CIPS/graphene heterostructure aligned on the PC-coated glass stamp is aligned to the target SiN waveguide. The sample is heated to 180 °C to release the PC film onto the SiN chip. The device is then soaked in chloroform (CF) to dissolve the PC, leaving a clean and well-transferred heterostructure positioned over the desired ring resonator, as shown in panel (3).

Figures S5–S7 illustrate the complete fabrication and transfer process used to realize a clean WS_2_/CIPS/graphene heterostructure on the SiN microring resonator. First, individual monolayer graphene, few-layer CIPS, and monolayer WSe_2_ flakes were exfoliated onto SiO_2_/Si substrates and identified by their optical contrast, as shown in Figure S5. Each selected flake was then sequentially picked up using a polycarbonate (PC) stamp mounted on a glass slide (Figure S6), beginning with graphene, followed by CIPS using controlled temperature to ensure proper adhesion, and finally WSe_2_ to complete the vertical stack. Once the full heterostructure was assembled, the PC/stack was carefully aligned to the pre-patterned SiN waveguide, and the sample was heated to 180 °C to release the PC film onto the chip (Figure S7). The device was subsequently submerged in chloroform to fully dissolve the PC layer, resulting in a clean and accurately transferred heterostructure positioned directly over the microring’s active region.

1. **Non-volatile phase modulation characteristics under different pulse conditions**


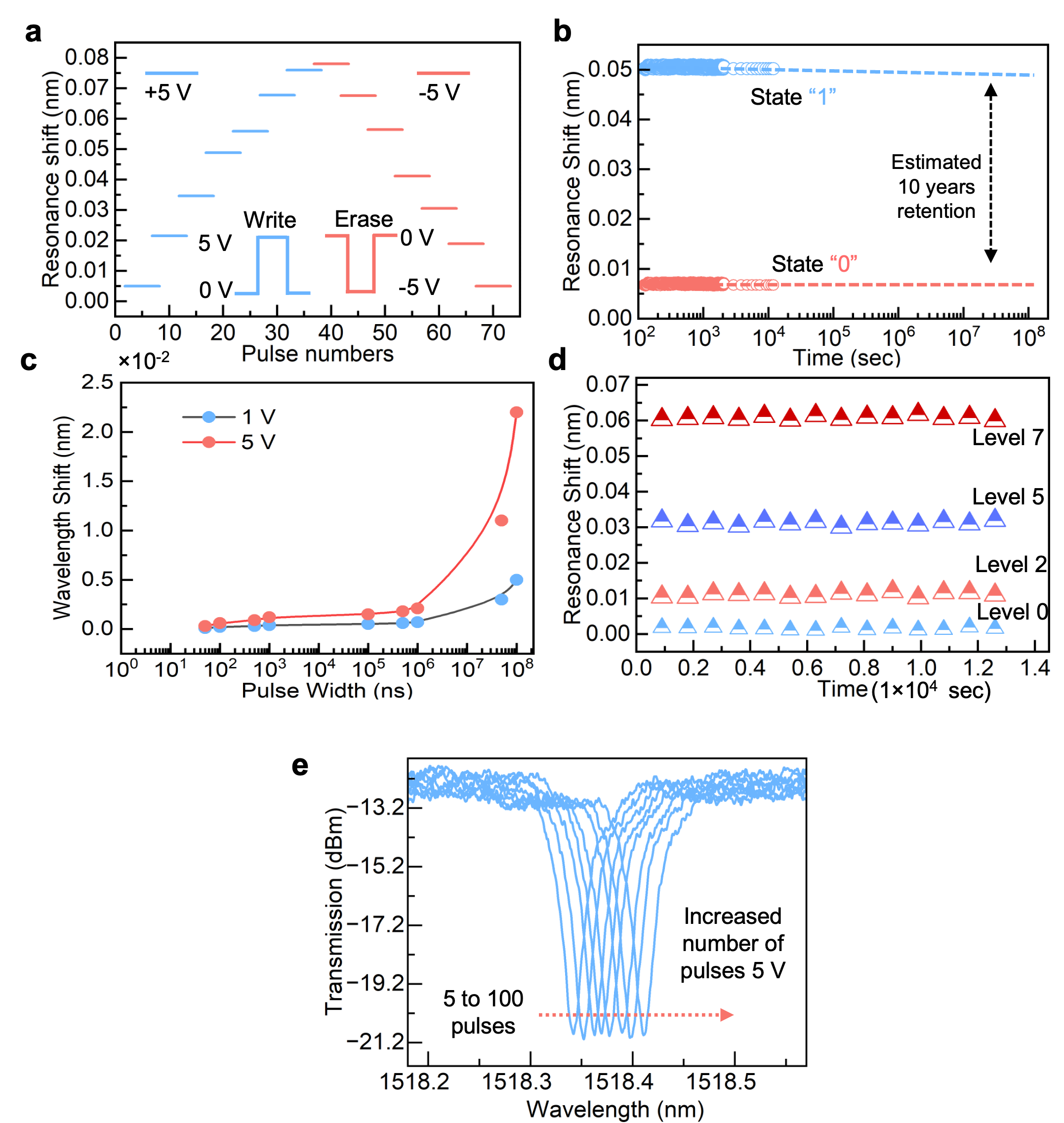


**Figure S8. Non-volatile phase modulation characteristics under different pulse conditions.** (a) Resonance wavelengths shift with increasing number of +5 V pulses, followed by reversal using pulses of -5 V. (b) Comparison of resonance positions in two bistable states and their retention over time. (c) Effect of varying pulse width on the magnitude of the resonance shift. (d) Retention characteristics at multiple intermediate states, showing multi-level stability. (e) Transmission spectra of the second device under repeated application of 5 V, 100 µs pulses, showing incremental resonance shifts.

1. **AFM images and Thickness calculations**


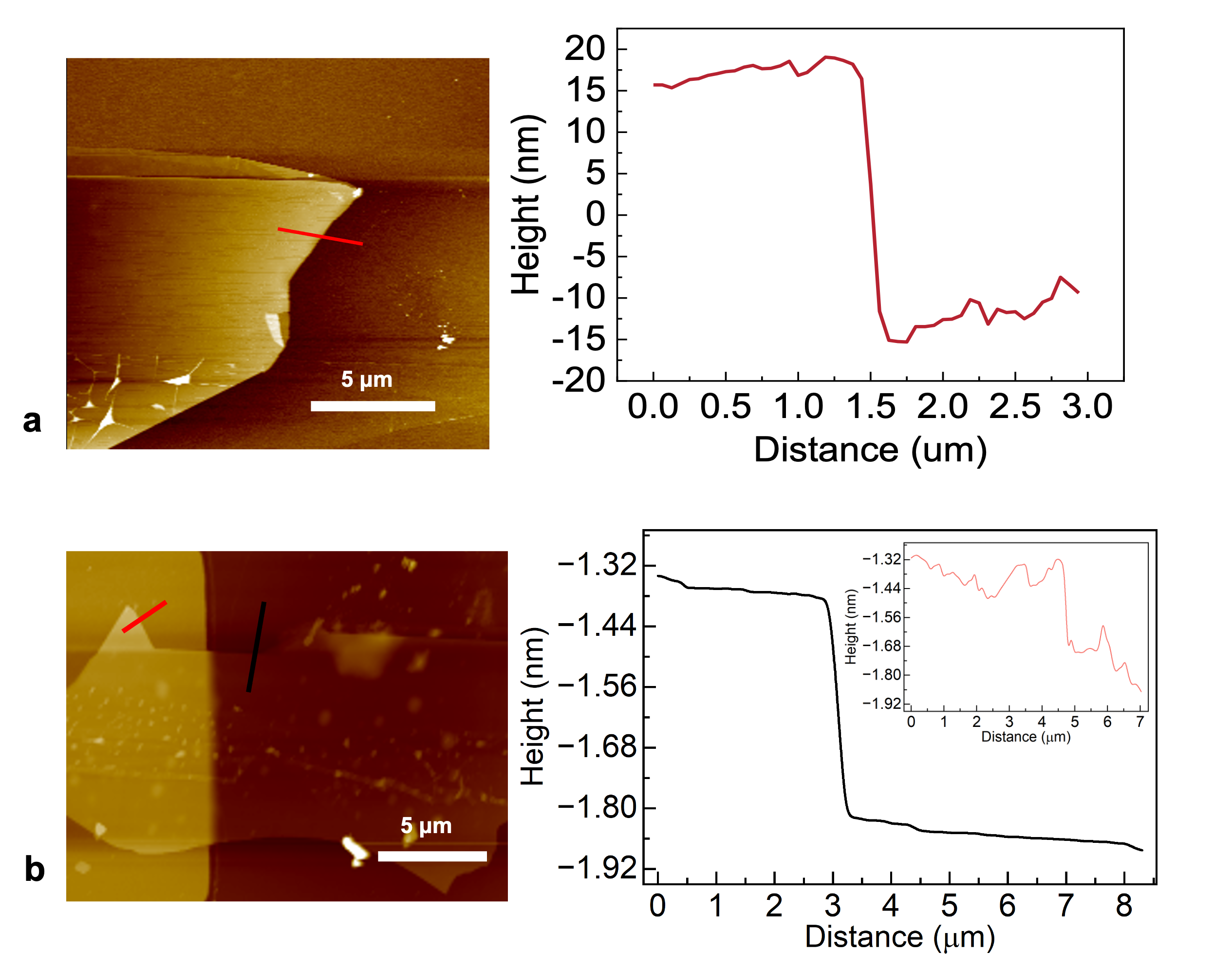


**Figure S9. AFM characterization of the heterostructure layers used in the phase modulator devices.** (a) Atomic force microscopy (AFM) scan showing the measured thickness of the CuInP_2_S_6_ (CIPS) layer for Device 1. (b) AFM image and corresponding height profile for Device 2, confirming CIPS thickness; inset shows the step height corresponding to the monolayer WS_2_ flake integrated on the same ring.

1. **Raman spectroscopy**

**
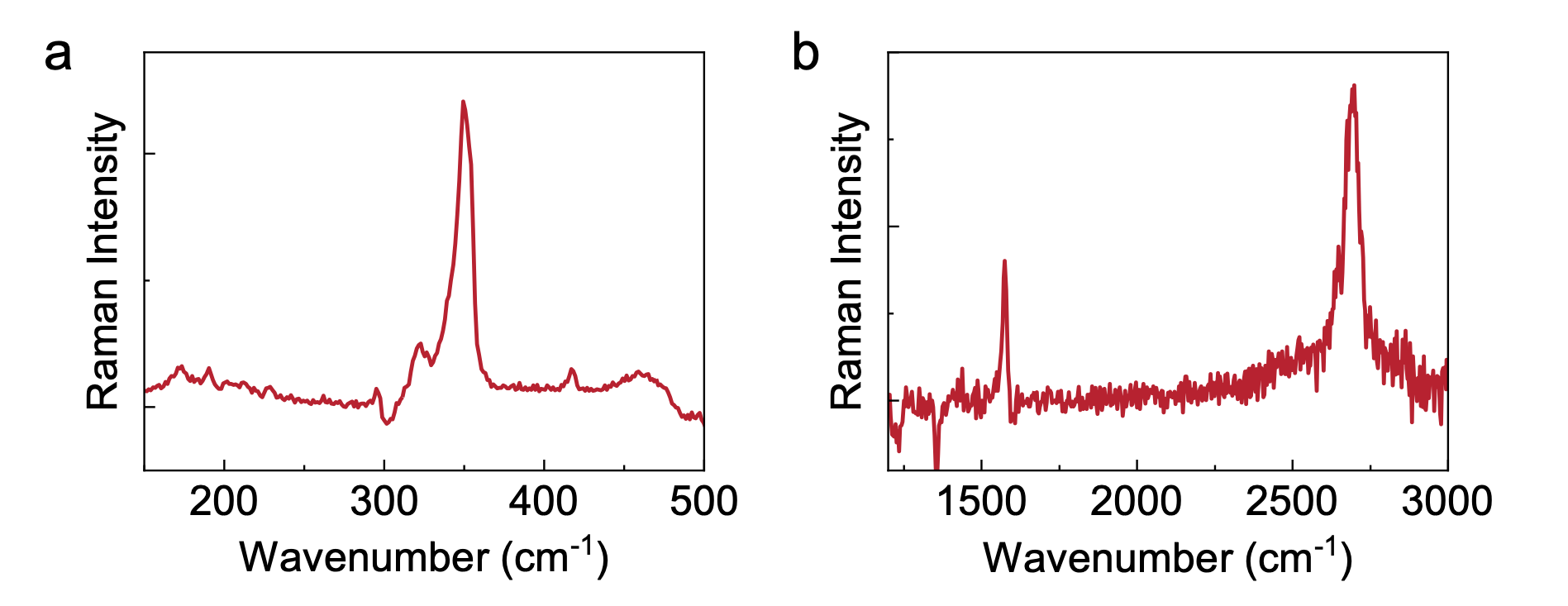
**

**Figure S10. Raman spectroscopy of the constituent monolayers used for heterostructure fabrication.** (a) Raman spectrum of monolayer WS₂, consistent with monolayer thickness, validating its suitability for device integration. (b) Raman shift spectrum of monolayer graphene, confirming high-quality single-layer graphene.

1. **PFM measurement**


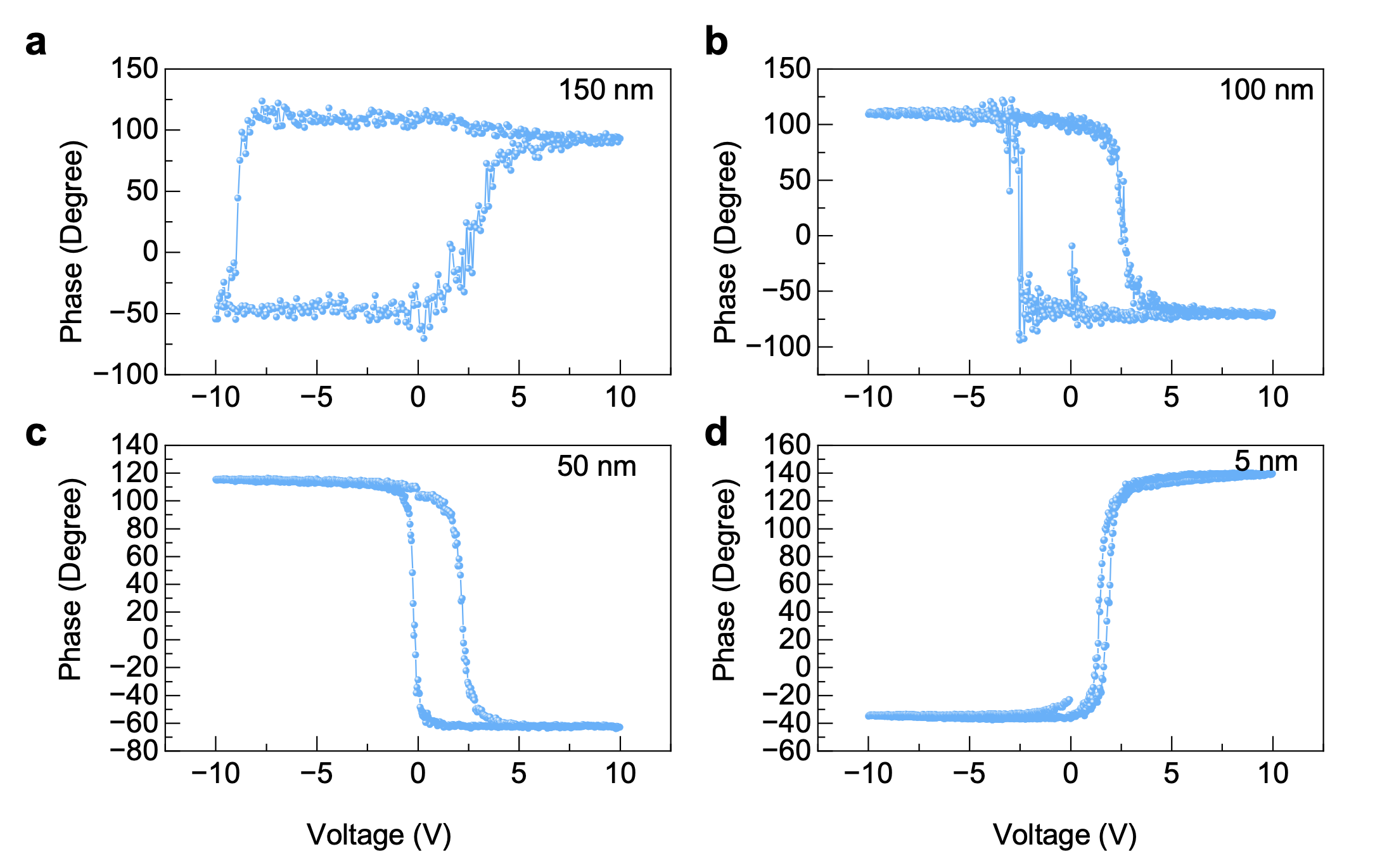


**Figure S11.** **To confirm ferroelectricity**: PFM measurements performed on different thickness of the CIPS flakes to confirm ferroelectric behaviour. (a) 150 nm thickness, (b) 100 nm, (c) 50 nm thickness and (d) 5 nm thickness. Multiple write–read cycles were carried out by applying a ±10 V bias at several points across flakes of different thicknesses, demonstrating consistent and repeatable ferroelectric switching.

**Reference**

1. Shusta, Prits, Guranich, Gerzanich, & Slivka. Dielectric properties of CuInP_{2}S_{6} crystals under high pressure. *Condens. Matter Phys.* **10**, 91 (2007).

2. Smidstrup, S. *et al.* QuantumATK: an integrated platform of electronic and atomic-scale modelling tools. *J. Phys. Condens. Matter* **32**, 015901 (2019).

3. Monkhorst, H. J. & Pack, J. D. Special points for Brillouin-zone integrations. *Phys. Rev. B* **13**, 5188–5192 (1976).

4. Perdew, J. P., Burke, K. & Ernzerhof, M. Generalized Gradient Approximation Made Simple. *Phys. Rev. Lett.* **77**, 3865–3868 (1996).

5. Grimme, S., Ehrlich, S. & Goerigk, L. Effect of the damping function in dispersion corrected density functional theory. *J. Comput. Chem.* **32**, 1456–1465 (2011).

6. Grimme, S., Antony, J., Ehrlich, S. & Krieg, H. A consistent and accurate *ab initio* parametrization of density functional dispersion correction (DFT-D) for the 94 elements H-Pu. *J. Chem. Phys.* **132**, 154104 (2010).

7. Zhou, S. *et al.* Van der Waals Layered Ferroelectric CuInP2S6: Physical Properties and Device Applications. Preprint at https://doi.org/10.48550/arXiv.2009.02097 (2020).

8. Wang, X. *et al.* Van der Waals engineering of ferroelectric heterostructures for long-retention memory. *Nat. Commun.* **12**, 1–8 (2021).

9. Ravindra, N. M., Ganapathy, P. & Choi, J. Energy gap–refractive index relations in semiconductors – An overview. *Infrared Phys. Technol.* **50**, 21–29 (2007).

10. Geler-Kremer, J. *et al.* A ferroelectric multilevel non-volatile photonic phase shifter. *Nat. Photonics* **16**, 491–497 (2022).
